# Chitin Attenuates Expression of *Listeria monocytogenes* Virulence Genes *in vitro*

**DOI:** 10.3389/fmicb.2020.588906

**Published:** 2020-12-03

**Authors:** Miguel Villoria Recio, Bo-Hyung Lee, Eva Maria Sternkopf Lillebæk, Birgitte H. Kallipolitis, Cormac G. M. Gahan, Hanne Ingmer, Marianne Halberg Larsen

**Affiliations:** ^1^Department of Veterinary and Animal Sciences, Faculty of Health and Medical Sciences, Food Safety and Zoonoses—University of Copenhagen, Frederiksberg, Denmark; ^2^Alimentary Pharmabotic Centre Microbiome Ireland, University College Cork, Cork, Ireland; ^3^School of Microbiology, University College Cork, Cork, Ireland; ^4^Université Paris-Saclay, INRAE, UVSQ, VIM, Jouy-en-Josas, France; ^5^Department of Biochemistry and Molecular Biology, University of Southern Denmark, Odense, Denmark

**Keywords:** chitin, listeria, PrfA, virulence, regulation

## Abstract

External signals are crucial for bacteria to sense their immediate environment and fine-tune gene expression accordingly. The foodborne pathogen *Listeria monocytogenes* senses a range of environmental cues in order to activate or deactivate the virulence-inducing transcriptional factor PrfA during transition between infectious and saprophytic lifecycles. Chitin is an abundant biopolymer formed from linked β-(1–4)-N-acetyl-D-glucosamine residues associated with fungi, the exoskeleton of insects and often incorporated into foods as a thickener or stabilizer. *L. monocytogenes* evolved to hydrolyse chitin, presumably, to facilitate nutrient acquisition from competitive environments such as soil where the polymer is abundant. Since mammals do not produce chitin, we reasoned that the polymer could serve as an environmental signal contributing to repression of *L. monocytogenes* PrfA-dependent expression. This study shows a significant downregulation of the core PrfA-regulon during virulence-inducing conditions *in vitro* in the presence of chitin. Our data suggest this phenomenon occurs through a mechanism that differs from PTS-transport of oligosaccharides generated from either degradation or chitinase-mediated hydrolysis of the polymer. Importantly, an indication that chitin can repress virulence expression of a constitutively active PrfA^∗^ mutant is shown, possibly mediated via a post-translational modification inhibiting PrfA^∗^ activity. To our knowledge, this is the first time that chitin is reported as a molecule with anti-virulence properties against a pathogenic bacterium. Thus, our findings identify chitin as a signal which may downregulate the virulence potential of the pathogen and may provide an alternative approach toward reducing disease risk.

## Introduction

*Listeria monocytogenes* is a ubiquitous foodborne pathogen and the causative agent of listeriosis, a serious disease that can be life-threatening in both humans and animals (Schlech and Acheson, 2000; Vázquez-Boland et al., 2001; de las Heras et al., 2011). Immunocompromised individuals are at higher risk and mortality rates can reach as high as 30% despite the use of appropriate treatment with antibiotics (Ramaswamy et al., 2007). Novel approaches are needed to interfere with bacterial virulence as an alternative way to combat listeriosis.

The pathogenicity of *L. monocytogenes* is largely determined by a series of virulence factors strongly induced inside the host and tightly regulated by the major virulence regulator PrfA (de las Heras et al., 2011). The core PrfA virulon is regulated at the transcriptional level from PrfA-dependent promoters and includes the pathogenicity island-1 (LIPI-1) genes, the *inlAB* operon and the *inlC*, *bsh*, and *uhpT* monocistrons (Scortti et al., 2007). Further regulation also involves the general stress response regulator σ^*B*^, direct binding of the nutrient-response regulator CodY to the *prfA* sequence in low branched-chained amino-acids availability, the environmental concentration of L-glutamine and a positive feedback loop dependent on cytosolic amounts of PrfA (Mengaud et al., 1991; Johansson et al., 2002; Scortti et al., 2007; de las Heras et al., 2011; Lobel et al., 2012; Haber et al., 2017; Lebreton and Cossart, 2017). At the post-translational level, PrfA activity is influenced by (i) the net balance of PrfA-activating glutathione, synthesized from exogenous cys-containing peptides, and the direct binding of PrfA-inhibiting non-cysteine-containing peptides (Reniere et al., 2014; Krypotou et al., 2019) and (ii) environmental availability of specific carbohydrates (Herro et al., 2006; Deutscher et al., 2014). Thus, PrfA facilitates transformation from the saprophyte to pathogen by integrating environmental cues that help fine-tune virulence gene expression accordingly (Freitag et al., 2009; Toledo-Arana et al., 2009).

Phosphotransferase system (PTS) transported carbohydrates contribute to virulence downregulation (Stoll and Goebel, 2010). The plant-derived cellobiose represses PrfA activity via the operon *bvrABC*, a beta-glucoside specific sensory system (Brehm et al., 1999; Vázquez-Boland et al., 2001; Scortti et al., 2007) and is highly repressive amongst the PTS-carbohydrates tested *in vitro* (Herro et al., 2006; Joseph et al., 2008). Other PTS-transported sugars, including glucose, contribute to repressing PrfA activity to various degrees by a complex mechanism that involves the phosphorylation of PTS components upon carbohydrate transport into the cell (Mertins et al., 2007; de las Heras et al., 2011). In contrast, PrfA activity repression is alleviated during growth on the non-PTS carbohydrate glycerol, enabling enhanced virulence gene expression (Joseph et al., 2008; Stoll et al., 2008). PTS-transported carbohydrates cannot be applied as potential anti-virulence molecules because they are readily metabolized by *L. monocytogenes* (Premaratne et al., 1991; Milenbachs et al., 1997). However, the effect of complex carbohydrates incapable of promoting rapid growth has not been previously examined as virulence inhibitors to our knowledge.

Chitin is a complex carbohydrate formed by linear chains of N-Acetylglucosamine (GlcNAc) residues that build a poorly soluble, high-strength matrix (Rinaudo, 2006). Present in the cell walls of fungi, fish scales, and the exoskeleton of animals (e.g., arthropods, crustaceans and molluscs), chitin is the second most abundant polymer on earth (Gooday, 1990). *L. monocytogenes* harbors a chitinolytic system formed by two chitinases (ChiA and ChiB) and the chitin-binding lytic polysaccharide monooxygenase LPMO10 (*lmo2467*) (Leisner et al., 2008). Chitinases are induced in soil and could presumably contribute to environmental survival by orchestrating hydrolysis of chitin into smaller units that can serve as carbon and nitrogen sources (Leisner et al., 2008; Piveteau et al., 2011; Paspaliari et al., 2015), likely the PTS-transported GlcNAc and chitobiose (Premaratne et al., 1991; Milenbachs et al., 1997). Whilst the polymer can extend *L. monocytogenes* survival in long periods of nutrient deprivation *in vitro*, no rapid growth on chitin occurs in minimal or rich media (Premaratne et al., 1991; Larsen et al., 2010).

We hypothesized that the polymer could serve as an environmental signal repressing virulence gene expression to maintain fitness during saprophytic existence. In the present study, the effect of chitin on PrfA-dependent expression was characterized and its mechanism of action explored during virulence-inducing conditions *in vitro*.

## Materials and Methods

### Bacterial Strains and Growth Conditions

*L. monocytogenes* cells were kept at −80°C in 25% glycerol vials and subcultured in brain heart infusion (BHI) agar (Oxoid, Hampshire, United Kingdom) plates at 37°C. Single colonies were used to inoculate BHI broth at 37°C (Oxoid). Overnight cultures were washed 3 times in saline solution (0.9% (v/v) NaCl) prior to bacterial transfer into a new media at a starting optical density 600nm (O.D_600_) of 0.05. *L. monocytogenes* EGD-e (serotype 1/2a) from Prof. Werner Goebel’s laboratory (Biozentrum, University of Würzburg, Germany) was employed for transcriptomic analysis. Cells were grown aerobically in 50 ml of media inside 250 ml flasks, at 37°C and 190 revolutions per minute (rpm). A chemically defined media (CDM) was prepared as previously described (Krypotou et al., 2019) except for carbon supplementation with glycerol (CDM_*gly*_), glucose (CDM_*glc*_) or cellobiose (CDM_*cellob*_) (Sigma-Aldrich, Darmstadt, Germany) at a concentration of 25 mM concentration with/without 0.2% (w/v) chitin unless otherwise stated. Bioluminescence assays were performed with an EGD-e strain containing the chromosome-integrative plasmid pPL2 with the *hly* promoter (P*_*hly*_*) fused to a *lux*ABCDE reporter (EGD-e::pPL2lux-P*_*hly*_*) (Bron et al., 2006). Chloramphenicol (7.5 μg/ml) was added when appropriate (Lauer et al., 2002; Bron et al., 2006). BHI supplemented with 0.2% activated charcoal (AC) (BHI_*AC*_) (Merck, Darmstadt, Germany) and Dulbecco’s Modified Eagle Medium (DMEM) (Sigma-Aldrich) with/without chitin were also used for virulence-inducing experiments. Haemolytic activity assays were conducted with strain EGD-e, its derivative mutant EGD-e Δ*chiA*Δ*chiB*, and an EGD G155S-PrfA^∗^ mutant (Lillebæk et al., 2017). This EGD G155S-PrfA^∗^ strain is FLAG-tagged in PrfA^∗^ N-terminal and was employed for Western blot experiments.

### Colloidal Chitin Preparation

Chitin (Sigma-Aldrich) was prepared with/without HCl treatment as previously described (Paspaliari et al., 2014), except that 24 ml of sterile distilled water was added. The resulting suspension of colloidal chitin was kept at 4°C.

### Total DNA and RNA Extraction, DNAse Treatment and Complementary DNA Synthesis

Chromosomal DNA from *L. monocytogenes* EGD-e was extracted from cells grown in BHI broth with DNeasy Blood and Tissue kit (Qiagen) as described in manufacturer’s instructions.

EGD-e cells were harvested at late exponential phase (O.D_600_ ≈ 1) and stationary phase (after 24 h incubation) and mixed 2:5 with bacterial RNAprotect Bacteria Reagent (Qiagen, Hilden, Germany) for 5 min at room temperature. Samples were then centrifuged at 4°C, 10,000 rpm and pellets were stored at −80°C until processed for RNA extraction. Bacterial pellets were mixed 1:100 β-mercapthoethanol/lysis buffer solution from RNAeasy mini kit (Qiagen) and lysed with a FastPrep-24 (MP Biomedicals, California, United States) instrument in 3 runs of 45 s with speed set at 6.0. The procedure followed the manufactorer’s instructions and included RNA-free DNAse treatment (Qiagen). Total RNA concentration and purity (ratio 260/280 and 260/230) was measured with a NanoDrop ND-1000 (Wilmington, DE, United States) spectrophotometer. RNA integrity based on 23S/16S band pattern was analyzed on a 1% agarose gel. Total RNA samples were stored at −80°C until further use.

To generate complementary DNA, 5 μL of pure total RNA were mixed with 2 μL of 10X Random Hexamer Primers (Thermo Fisher Scientific, MA, United States) and incubated at 65°C for 5 min. A final volume of 20 μL containing 7 μL of the previous mixture, 4.6 μL of Nuclease-Free MilliQ Water (Thermo Fisher Scientific), 1 μL of dNTPs, 4 μL of 5X ImProm-II buffer and 1 μL of Reverse Transcriptase (Promega, Wisconsin, United States) was subjected to the following thermal cycling parameters; incubation at 25°C for 5 min, reverse transcription reaction at 42°C for 60 min, enzymatic inactivation at 70°C for 15 min and hold at 4°C. The final mixture was diluted 1:10 with Nuclease-Free MilliQ Water (Thermo Fisher Scientific).

### Real Time—Quantitative Polymerase Chain Reaction (RT-qPCR)

Quantification and normalization of *actA* expression levels were performed as previously described (Krypotou et al., 2019), with *rpoB* and *ldh* as housekeeping genes (Deshayes et al., 2012). However, primers and probe sequences and concentrations differed (Supplementary Table S1). Cycling parameters included a preincubation of TaqGold polymerase for thermal activation at 95°C for 10 min and a 2-step amplification comprising 40 cycles of 15 s of denaturalization at 95°C and 60 s of annealing/extension at 65°C for all genes. Increase/decrease temperature ramp ratio was performed at 4.4 and 2.2°C/s, respectively. The reported fluorescence was acquired after every cycle and the reaction was then held at 4°C.

### RNA Sequencing (RNA-Seq) and Analysis

The following procedure was performed by the company GenXPro (Frankfurt, Germany) as follows; ribosomal RNA (rRNA) depletion (mRNA enrichment) from total RNA was performed as instructed using an rRNA removal kit (Illumina, San Diego, CA). Enriched mRNA was purified by Zymo-Spin column (ZymoResesarch, Irvine, CA) and run on Labchip GX II bioanalyzer (Perkin Elmer, Waltham, MA) to confirm reduction of rRNA. The preparation of cDNA fragment libraries and RNA-Seq was performed using the NEBNext^®^ Ultra^TM^ II Directional RNA Library Prep Kit for Illumina^TM^ (Illumina, Ipswich, MA), except that the enriched mRNA was fragmented for 15 min at 94°C and reverse transcribed to synthesize the first-strand cDNA followed by second strand cDNA synthesis. Double-stranded cDNA (ds cDNA) was purified using NucleoMag (Macherey nagel, Düren, Germany) SPRI selection. End repair was performed on the ds-cDNA library followed by ligation of adaptors. After purification using NucleoMag SPRI beads, test qRT-PCR (Applied Biosystems) was performed using KAPA Hifi polymerase (Roche) with EvaGreen^®^ (Biotium, Fremont, CA) to determine appropriate cycle numbers for PCR. High fidelity PCR was performed using KAPA Hifi polymerase (Wilmington, MA, United States) with NEBNext Multiplex Oligos for Illumina (Dual Index Primers) to selectively enrich library fragments. The PCR products were purified twice using NucleoMag (Macherey nagel) SPRI beads and the quality of the final library was assessed on Labchip GX II bioanalyzer (Perkin Elmer). The libraries were sequenced on an Illumina NextSeq 500 platform (Illumina) (paired-end, 2 X 75 bp per read). Sequencing quality was assessed using “FastQC,” and Illumina adapter sequences and low-quality base pairs were removed using cutadapt version 1.9 (Martin, 2011). Reads were mapped to the complete sequenced genome of reference strains EGDe (ENSEMBL ASM19603v1) using Bowtie 2 v 2.2.4 with standard parameters and sensitive-local (Langmead and Salzberg, 2012). BAM alignment files were used as input for read counting using htseq-count version 0.6.0. Differential expression (DE) analyses were performed using DESeq2 in “R” v 3.2.2 (Love et al., 2014), and the DE was reported as log2 fold changes. *p*-values were adjusted by the DESeq2 default Benjamini-Hochberg adjustment method and genes with a > twofold (> 1 log2) change in expression and an adjusted *p*-value < 0.05 were reported as DE. All deferentially expressed genes (DEGs) were analyzed on Gene Ontology (GO) enrichment and Kyoto Encyclopedia of Genes and Genomes (KEGG) for function and pathways and those with a cut off with fold change (FC) ≤ −1 or ≥ 1 are shown in Supplementary Tables S2, S3, respectively.

### Haemolytic Activity

The quantification of listeriolysin O (LLO) activity present in culture supernatants after growth in CDM_*gly*_ and CDM_*gly + chitin*_ was performed as previously described in Larsen et al. (2006) and haemolytic activity was expressed in percentage as shown in Dacheux et al. (2001). CDM was used as negative control and cells were treated with 0.1% triton X-100 (Sigma-Aldrich) as positive control (100% haemolysis).

### *In vitro* Bioluminescence Imaging

Phenotypic imaging was performed on EGD-e::pPL2lux-P*_*hly*_* grown in CDM_*gly*__/glc_ or BHI_*AC*_ with/without chitin. Cultures were transferred to a Costar 6-well plate (Sigma-Aldrich) and imaged for bioluminescence measurement with an IVIS 100 system (Xenogen, CA, United States) chamber.

### Preparation and Analysis of Extracellular Proteins by Western Blot

*L. monocytogenes* grown in CDM_*gly*_ and BHI, BHI-AC with and without 0.2% colloidal chitin were collected in late exponential phase (O.D_600_ = 1 and 2) and stationary phase (after 24 and 5 h), respectively. Cultures where centrifuged 8,000 rpm, 5 min and supernatant was discarded. Pellets were stored at −80°C until further use. When needed, pellets were lysed and the resulting protein extract was stored at −20°C. PrfA and ActA detection was performed as previously indicated (Lillebæk et al., 2017).

### Statistical Analysis

All experiments were performed in technical duplicates and in three independent biological replicates. The statistical significance was assessed by performing Student’s *t*-tests on GraphPad Prism software (San Diego, CA, United States) and significance considered as ^∗^*p* < 0.05; ^∗∗^*p* < 0.01; ^∗∗∗^*p* < 0.005; ^****^p < 0.0001.

## Results

### *L. monocytogenes* Transcriptional Profile During Growth *in vitro* in the Presence of Chitin

**FIGURE 1:**
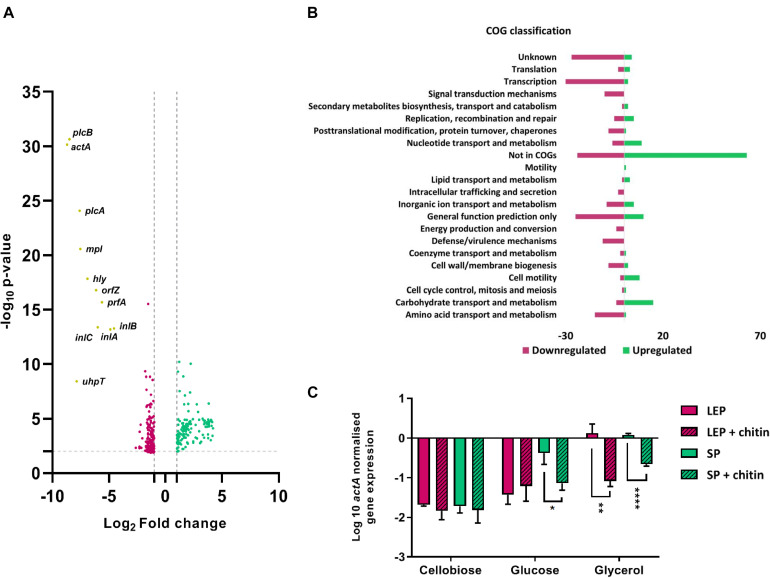
**(A)** Volcano plot comparing DEGs of *L. monocytogenes* EGD-e grown in CDM_*gly*_ to CDM_*gly* + chitin_ in late exponential phase. Upregulated DEGs are represented in red and downregulated in green. Non-significantly expressed genes were excluded by applying an FDR < 0.05 and a log2-fold change (FG) of ≤ *−*1 and ≥ 1. Core PrfA-regulon genes are downregulated and highlighted in yellow. **(B)** DEGs distributed in COG functional categories with numbers of downregulated genes in red and upregulated in green. **(C)** qRT-PCR absolute quantification by geometric mean (*rpoB/ldh*) showing the effect of chitin in *actA* expression in different carbon sources in CDM. Chitin represses significantly *actA* in late exponential phase (LEP) in CDM_*gly*_ and in stationary phase (SP) in both CDM_*gly*_ and CDM_*glc*_. Error bars stand for standard deviation.

The transcriptional profile of *L. monocytogenes* grown under virulence inducing conditions was compared with/without chitin (CDM_*gly* + chitin_/CDM_*gly*_) using RNA-Seq to assess the effect of chitin on virulence expression. A total of 339 DEGs were identified, of which 137 were upregulated and 202 downregulated in response to chitin (Figure 1A). When DEGs were classified by clusters of orthologs groups (COGs), upregulated genes mainly involved uncategorized functions and transport and metabolism, including carbohydrate, nucleotide, inorganic ion and lipid, and cell motility (Figure 1B and Supplementary Table S2). The most abundant COG categories under repression were transcription, amino acid and inorganic ion metabolism and transport, defense/virulence mechanisms, signal transduction mechanisms and cell wall membrane/biogenesis (Figure 1B). Downregulated DEGs included PrfA-dependent virulence genes, including the operon *inlAB* (*lmo0433*-*0434*), the monocistron *uhpT* (*lmo0838*) and all LIPI-1 genes with a log2 FC ≥ 4.5 and a false discovery rate (FDR) < 0.0001 (Figure 1A and Supplementary Table S3).

The expression of several non-coding RNAs (ncRNAs) associated with infection was also regulated in the presence of chitin (Supplementary Table S4). Rli47, previously shown to be induced in the intestine and macrophages (Toledo-Arana et al., 2009; Mraheil et al., 2011) was found induced by chitin with a log2 FC ≈ 5.3 and an FDR = 0.001. The ncRNA Rli17 (LhrA) is a negative intermediator partially regulating the positive control of the Agr sytem over *chiA* (Nielsen et al., 2011; Paspaliari et al., 2014). *Agr* is induced in stationary phase in response to addition of chitin (Nielsen et al., 2011). In our comparison, LhrA was upregulated by chitin at a log2 FC ≈ 2.7 and an FDR < 0.001. Amongst others, Rli74 was the most downregulated ncRNA by chitin, with a log2 FC of ≈−9 and an FDR < 0.001 (Supplementary Table S4). Rli74 is believed to be the 5’UTR of the virulence gene *actA* (Mellin and Cossart, 2012; Lebreton and Cossart, 2017).

In order to confirm PrfA-dependent repression, expression levels of the strictly PrfA-dependent gene *actA* (de las Heras et al., 2011) was assessed under the same conditions by qRT-PCR. Notably, *actA* was significantly downregulated in CDM_*gly* + chitin_ in both late exponential phase and stationary phase in comparison to CDM_*gly*_ (∼17 and 3.2 times less, respectively) (Figure 1C). Chitin mediated repression was also observed at 30°C, where *actA* upregulation in CDM_*gly*_ in late exponential phase was significantly downregulated in the presence of chitin (∼28 times less) (Supplementary Figure S1).

To further characterize the strength of the chitin repressor effect, *actA* gene expression levels were compared to those of bacteria growing in two PTS-transported sugars known to repress virulence genes expression (glucose and cellobiose) with/without chitin. At 30°C, the expression of *actA* was low in both sugars and the presence of chitin did not lead to further repression. However, expression levels increased in stationary phase at 37°C in CDM_*glc*_ and significant repression was found when chitin was present (∼6 times less) (Figure 1C).

### Chitin Titration on P*_*hly*_*-Dependent Bioluminescence and LLO Expression

**FIGURE 2:**
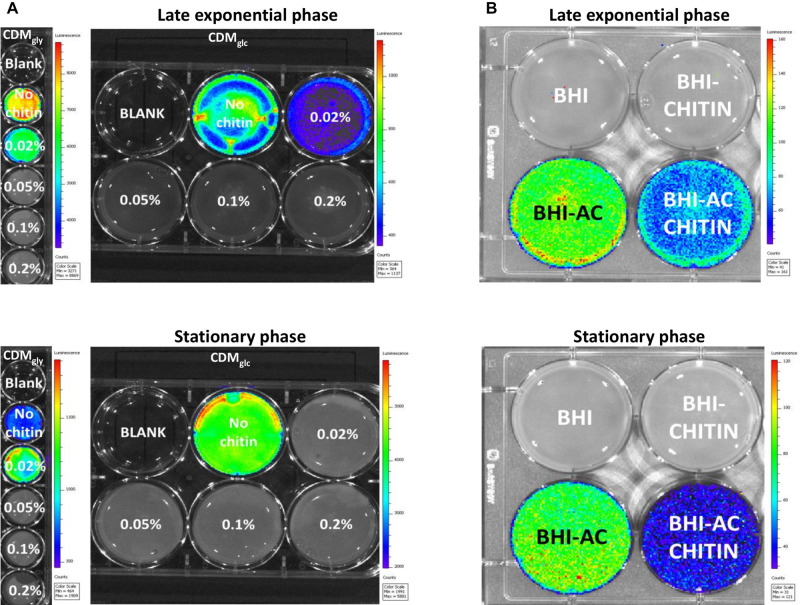
Bioluminescence images of P*_*hly*_*-dependent expression in an EGD-e::pPL2*lux*-P*_*hly*_* mutant. Bioluminescence is color-coded and represents high light emission intensity in red and low in blue. **(A)** Chitin downregulates P*_*hly*_*-dependent expression down to a concentration of 0.05% during growth in both CDM_*gly*_ and CDM_*glc*_ both late exponential phase and stationary phase. Integration time was 1 min in late exponential phase for both CDM_*gly*_ and CDM_*glc*_ and 2 min in CDM_*gly*_ and 1 min in CDM_*glc*_ in stationary phase. **(B)** The effect of chitin during growth in BHI and virulence activating BHI_*AC*_, where chitin represses P*_*hly*_* expression in both late exponential and stationary phase. Images were obtained after 3 min of exposure.

**FIGURE 3:**
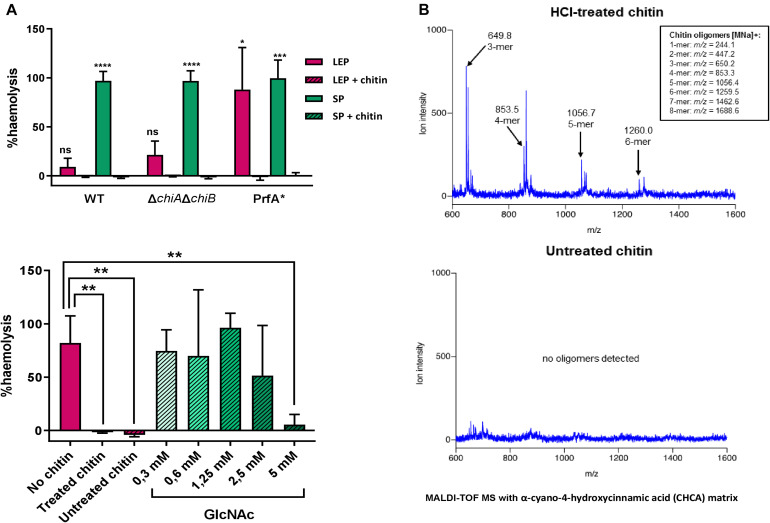
**(A)** Haemolytic activity generated by the LLO present in supernatants of *L. monocytogenes* cultures grown in CDM_*gly*_. Above, chitin represses haemolysis in stationary phase (SP) in a WT strain, and a Δ*chiA*Δ*chiB* mutant and in both late exponential phase (LEP) and SP in a PrfA^∗^ mutant. Below, comparison of the effect of HCl-treated chitin, untreated chitin and a gradient of GlcNAc on haemolytic activity in a WT strain. Both HCl-treated and untreated chitin can significantly repress haemolytic activity and a concentration of 5 mM GlcNAc is required to achieve the same repression. Data was normalized to a full haemolysis positive control and expressed in percentage. **(B)** Chromatographic identification and determination of chitin-derived particles present in an HCl-treated chitin suspension (above) and untreated chitin suspension (below) by MALDI-TOF MS. Whilst 3-mer and longer oligosaccharide chains were found in low concentrations in the HCl-treated, none were detected when chitin was left untreated.

A bioluminescent reporter strain allowing the quantification of strictly PrfA-dependent P*_*hly*_*-derived induction (EGD-e::pPL2lux-P*_*hly*_*) (Bron et al., 2006) was used to determine the minimum concentration at which chitin represses virulence expression. The virulence gene *hly* encodes a pore-forming hemolysin known as Listeriolysin O (LLO) (Schnupf and Portnoy, 2007). Chitin titration on bacteria growing in CDM_*gly*_ showed that expression from P*_*hly*_* was higher in late exponential phase than stationary phase, but significant repression by chitin was observed down to a concentration of 0.05% in either phase (Figure 2A). Chitin-mediated repression during growth in PTS-transported carbohydrates was also assessed in CDM_*gl*__*c*_ and CDM_*cellob*_. Here, no P*_*hly*_*-derived bioluminescence was generated while growing in CDM_*cellob*_, but chitin downregulated P*_*hly*_* expression in both late exponential phase and stationary phase down to a concentration of 0.05% in CDM_*glc*_ (Figure 2A). In line with this finding, bioluminescence of overnight cultures of EGD-e::pPL2lux-P*_*hly*_* was also downregulated by chitin in a glucose-supplemented cell culture media (Supplementary Figure S2). To confirm *hly* downregulation by chitin, the haemolytic activity produced by free LLO in the supernatant of EGD-e cultures was quantified. Here, LLO activity decreased in the presence of chitin in late exponential phase but was significant in stationary phase only (*p* < 0.0001) (Figure 3A).

### Chitin-Derived Oligosaccharides Are Not Responsible of Virulence Repression

To investigate whether chitin-derived oligosaccharides from chitin preparation were present in the media, the molecules in both HCl-treated and untreated chitin suspensions were identified using matrix assisted laser desorption ionization-time of flight mass spectrophotometry (MALDI-TOF MS). Neither the monomer GlcNAc or the dimer chitobiose were detected, but 3-mer, 4-mer and bigger oligomers were found in HCl-treated chitin (Figure 3B). Whether the presence of these oligosaccharides was responsible for virulence gene repression was assessed by studying the effect of untreated chitin on *L. monocytogenes* haemolytic activity in CDM_*gly*_ in comparison to that of HCl-treated chitin. Here, haemolytic activity was repressed in both HCl-treated and untreated chitin, indicating that the oligosaccharides present in HCl-treated chitin do not play a key role in chitin-mediated repression (Figure 3A). To determine the concentration at which free oligosaccharides start repressing PrfA-dependent *hly* expression, haemolytic activity on an increasing concentration gradient of GlcNAc was assessed. Whilst large variation across different concentrations was found, a concentration of 5 mM was required to significantly repress haemolytic activity (Figure 3A).

The role of chitinases in chitin-mediated gene repression was assessed to clarify whether enough GlcNAc or chitobiose to support growth were generated from chitin hydrolysis in CDM_*gly* + chitin_. To this end, haemolytic activity was assessed for EGD-e Δ*chiAchiB*, a double mutant unable to hydrolyse chitin (Leisner et al., 2008). Here, no haemolytic activity was detected in the presence of chitin in stationary phase in EGD-e Δ*chiA*Δ*chiB* either (Figure 3A).

### PrfA^∗^ Activity Could Be Inhibited by Chitin in CDM

It is unknown whether downregulation of *prfA* levels in our RNA-Seq experiments occurs at the transcriptional level or due to a post-translational modification of PrfA. Virulence genes are derepressed in CDM_*gly*_, but higher expression levels, similar to those seen during infection, can be reached by constitutively activating PrfA (PrfA^∗^). The active-locked PrfA conformation of this mutant leaves PrfA-dependent gene expression mainly conditioned to its activity in our growth conditions. A FLAG-tagged EGD G155S-*prfA*^∗^ mutant was therefore used to compare the levels of PrfA and the strictly PrfA-dependent ActA proteins with/without chitin in order to shed light on the chitin repressor effect phenomenon. Chitin repressor effect on virulence gene expression in this mutant was firstly assessed by quantifying haemolytic activity in CDM_*gly*_ with/without chitin. As expected, haemolysis was stronger in the G155S-*prfA*^∗^ mutant when compared to a WT EGD-e background. Indeed, chitin inhibited heamolytic activity completely in the G155S-*prfA*^∗^ mutant also (Figure 3A).

**FIGURE 4:**
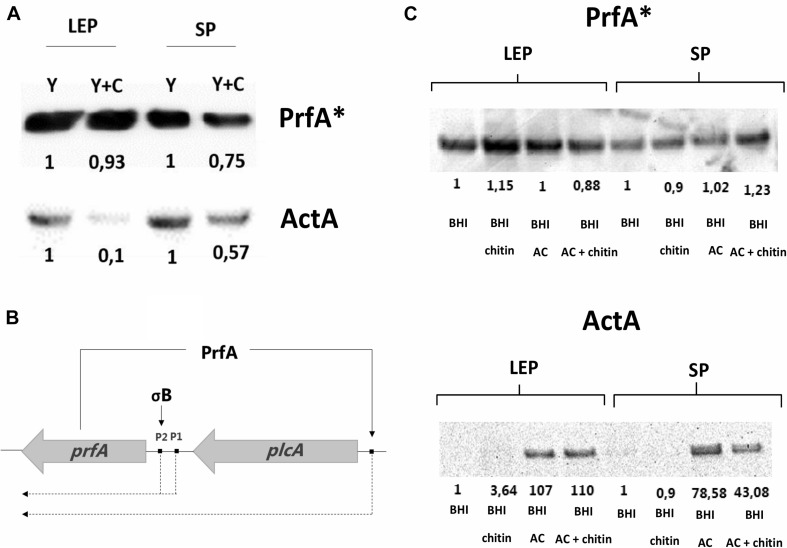
**(A)** Western blot analysis showing PrfA* and ActA levels generated by EGD G155S-PrfA* in CDM_*gly*_ = Y and CDM_*gly* + chitin_ = Y + C. PrfA* levels are weakly diminished in the presence of chitin, whilst ActA levels are reduced in SP and more significantly in LEP. The protein levels are relative to Y in each phase. **(B)** Representation of PrfA positive feedback loop. PrfA positively regulates its own expression by activating the synthesis of the *plcA*-*prfA* bicistron transcript and is necessary for full expression of PrfA-dependent genes. **(C)** PrfA* and ActA amounts in BHI and BHI_*AC*_ with/without chitin in LEP and SP. PrfA* levels are evenly distributed in the replicates whilst ActA levels increase in BHI_*AC*_ and are reduced by chitin in SP only. Here, protein levels are compared to BHI in each phase.

Western blot experiments showed that PrfA levels were marginally reduced if chitin was present in both late exponential and stationary phase, whilst ActA levels were strongly reduced by chitin in late exponential phase and only by half in stationary phase (Figure 4A). These results suggest that transcription of *prfA* still occurred from P1 and P2 promoters due to σ^*B*^ activity during growth in glycerol (Abram et al., 2008) and may not reach full expression levels because the PrfA activity-dependent feedback loop promoter is not induced (Figure 4B). In line, a stronger repression of ActA production happens in the presence of chitin, suggesting that a post-translational modification hindering PrfA^∗^ activity could occur.

### Chitin Can Repress AC-Mediated Induction of PrfA^*WT*^-Dependent Expression but May Not Inhibit PrfA^∗^ Activity in Rich Media

To further investigate chitin repressor effect, we employed a virulence-inducing mechanism that differs from that of non-PTS transported glycerol in *L. monocytogenes* (Ermolaeva et al., 2004). Briefly, AC is thought to hijack a diffusible auto-repressor excreted during growth in BHI, alleviating virulence downregulation at host body temperature (Ermolaeva et al., 2004). A bioluminescence assay was performed in order to quantify P*_*hly*_* expression in an EGD-e::pPL2lux-P*_*hly*_* mutant in the presence of chitin in BHI_*AC*_. No bioluminescence signal was detected in BHI alone or BHI_*chitin*_ (Figure 2B) but the signal increased when bacteria grew on BHI_*AC*_. Expression in BHI_*AC*_ was significantly downregulated in the presence of chitin (BHI_*AC* + chitin_) in both late exponential phase and stationary phase (Figure 2B).

Virulence expression was studied in the FLAG-tagged EGD G155S-*prfA*^∗^ mutant to elucidate whether the chitin repressor effect also occurred in this background in BHI_*AC*_. First, the LLO levels were measured by performing a haemolytic activity assay. Here, no significant repression was observed when BHI_*AC*_ was compared to BHI_*AC* + chitin_ (Supplementary Figure S3). To further explore the effect of chitin in BHI_*AC* + chitin_, PrfA^∗^ and ActA protein levels were compared. Western blot analysis showed that PrfA^∗^ levels remained consistent regardless of growth phase and media. ActA appeared almost undetectable in both late exponential phase and stationary phase in BHI and BHI_*chitin*_ and strongly induced in BHI_*AC*_. The low detection levels in BHI made it difficult to discern whether chitin could further repress ActA levels, but lower amounts were detected in stationary phase in BHI_*AC* + chitin_ in comparison to BHI_*AC*_ (Figure 4C).

## Discussion

Downregulating *prfA* expression or inhibiting PrfA activity has emerged as a new mechanism to combat listeriosis and prevent or enhance the use of antibiotics (Good et al., 2016; Dickey et al., 2017; Johnson and Abramovitch, 2017; Kulen et al., 2018). For example, essential oil from *Cannabis sativa* downregulates *prfA* and flagellar motility genes (*motA* and *motB*) during growth *in vitro* at 30°C and decreases bacterial invasion in Caco-2 cells (Marini et al., 2018). Besides, exposure to 6-N-hydroxylaminopurine reduces PrfA levels and activity, whilst ring-fused 2-pyridone binds to PrfA decreasing its affinity to the PrfA-binding site (Good et al., 2016; Krajewski et al., 2017; Kulen et al., 2018). Both effects result in repression of virulence expression, however, neither compound bypasses the effect of a constitutively active G145S-*prfA*^∗^ allele (Vega et al., 2004; Good et al., 2016; Krajewski et al., 2017; Kulen et al., 2018). In contrast, antimicrobial medium- and long-chain free fatty acids (FFAs) prevent the expression of PrfA-dependent genes, a repression that can overcome the effect of a mid-level constitutively active EGD G155S-*prfA*^∗^ mutant (Lillebæk et al., 2017). The repressor mechanism occurs by free fatty acids interfering with the DNA binding activity of PrfA (Dos Santos et al., 2020). Similarly, our studies showed that the chitin polymer repressed PrfA-dependent expression in both a WT strain and a G155S-*prfA*^∗^ mutant and suggest that a post-translational modification of PrfA^∗^ may inhibit the activity of the protein in CDM.

While the precise mechanism by which chitin represses virulence factor expression remains unknown, 7 PTS-system associated genes were upregulated in our RNA-seq analysis, but no genes linked to the metabolism of chitin-derived oligosaccharides, including the chitobiose-induced *lpo* operon (*lmo1718-1721)* (Dalet et al., 2003; Toledo-Arana et al., 2009) or the GlcNAc metabolism associated operon *lmo0956-0958* and *lmo2108* (Popowska et al., 2012) were found differentially expressed in response to chitin. Our results indicate that the chitin polymer is the molecular form responsible of virulence gene repression and that the inhibition of PrfA activity in our experimental setting is not mediated via PTS-transport-mediated signaling. Instead, the recognition of the chitin polymer by *L. monocytogenes* is likely to represent a niche-specific adaptation strategy. The event could generate a fine-tuning transcriptional response to adapt to the surrounding milieu in chitin-containing environments such as soil, resulting in virulence gene repression (Piveteau et al., 2011; Vasanthakrishnan et al., 2015).

Two-component systems are mechanisms that bacteria employ to sense and respond to changing environments, where a histidine kinase (HK) senses external stimuli and phosphorylates a response regulator (RR) that subsequently triggers a signaling cascade into the cell, often including DNA binding (Zschiedrich et al., 2016). The two-component system VirRS contributes significantly to *L. monocytogenes* virulence and controls global regulatory modifications of cell surface components (Mandin et al., 2005). Genes previously designated as positively regulated by the RR VirR (Mandin et al., 2005; Chatterjee et al., 2006; Shin et al., 2010; Grubaugh et al., 2018) were significantly downregulated in response to chitin in the RNA-Seq data. In addition, genes described as positively regulated by the HK VirS were also found to be downregulated, including *plcA*, *uhpT*, *lmo1251* (similar to PrfA). These data suggest an inverse relationship between the VirRS-inducible regulon and that of chitin and may indicate an inhibition of VirRS-dependent expression by chitin. The sensor HK *lmo1508* and the orphan RR DegU, previously shown to contribute to infection (Knudsen et al., 2004; Gueriri et al., 2008) were also found to be significantly repressed by chitin in our study.

In addition to two-component systems, a series of putative regulators associated with virulence and defense were found to be significantly repressed by chitin in our RNA-Seq data. Amongst these were the transition-state regulator antibiotic resistance protein B (AbrB) (Hamon et al., 2004), a series of deoxyribonucleoside (DeoR)-type family of transcriptional regulator genes and a LysR transcriptional regulator. LysR negatively regulates genes associated with flagellar motility (Abdelhamed et al., 2020), which are important for adhesion to abiotic surfaces and biofilm formation (Lemon et al., 2007). In agreement, our dataset showed an upregulation of flagellar motility genes, thus correlating with LysR downregulation. This finding suggests that chitin may over-ride both DegU- and temperature-dependency of flagellar expression (Peel et al., 1988; Williams et al., 2005) and suggests that flagella may be necessary to aid bacteria adherence to chitin particles in the natural environment. Other transcriptional regulators downregulated by chitin include the cell envelope-associated LytR type regulators (Hübscher et al., 2008), a multiple antibiotic resistance regulator MarR, the repressor of multidrug resistance elements TetR and AcrR, a series of genes assigned as GntR transcriptional regulators (Wassinger et al., 2013; Alonso et al., 2014) and the transcriptional regulator XRE (Hu et al., 2019). These data demonstrate that chitin induces major transcriptional reprofiling in *L. monocytogenes* influencing virulence/defense mechanisms, cell wall functions and signaling transduction.

Further research is required to elucidate the precise mechanisms by which chitin impacts virulence gene repression through inhibition of PrfA^*WT*^ and PrfA^∗^ activity in CDM and PrfA^*WT*^ in BHI. A recent study showed that exogenous exploitation of environmental oligopeptides scavenged via the Opp transport system allows *L. monocytogenes* to sense the surrounding milieu and modulate PrfA activity accordingly (Krypotou et al., 2019). The GshF enzyme, responsible of glutathione synthesis, is essential for both PrfA-dependent *actA* and *hly* genes expression during growth in both CDM and virulence activating XAD4 pre-treated BHI (Krypotou et al., 2019). In our RNA-Seq data, *gshF* was found to be significantly repressed by chitin in CDM_*gly*_, along with 15 genes associated with amino acid metabolism and transport, including a putative oligopeptidase f (*pepF*). Clarifying whether chitin exerts an effect on glutathione synthesis and the nature of virulence-repressive molecules absorbed by activated charcoal in BHI could aid in determining why PrfA^*WT*^ and PrfA^∗^ activity can be presumably inactivated by chitin in CDM and only PrfA^*WT*^ in BHI-AC.

The chitin polymer exerts beneficial properties in the gastrointestinal tract that in combination with a potential inhibition of PrfA activity, could aid in preventing listeriosis. There is evidence that chitin microparticles are capable of positively influencing gut-dwelling microbiota diversity in mice (Nagatani et al., 2012), which form a commensal barrier against pathogenic bacteria and generate metabolites that can improve the intestinal barrier function (Kelly et al., 2015). Many studies have demonstrated that chitin and chitin-containing microorganisms can trigger a series of innate and adaptive immune responses in the mammalian host by triggering pattern recognition receptors (Reese et al., 2007; Lee, 2009; Nagatani et al., 2012; Kim et al., 2015; Roy et al., 2019). Thus, dietary intervention with chitin could influence the activation of the immune system and subsequent immune responses against pathogenic bacteria. A recent publication linked high-fat diets (HFD) with an increased susceptibility to *L. monocytogenes* infection in mice (Las Heras et al., 2019). Interestingly, a separate study showed that chitin oligosaccharide inhibit the destruction of the gut barrier in HFD-treated mice, positively influenced the growth of beneficial bacteria and reduce the abundance of inflammogenic species (Zheng et al., 2018). The combination of a prebiotic effect, immune system response modulation and an *in vitro* anti-virulence activity makes chitin an excellent candidate to investigate listeriosis prevention in the context of infection. These future studies will be crucial in order to improve our understanding on how the polymer could contribute to prevention of listeriosis.

## Data Availability Statement

The raw and processed data of the RNA-Seq analysis supporting the findings of this study were generated at GenXpro, Frankfurt, Germany and will be made available from the corresponding author on request to any qualified researcher. Data have been uploaded to Gene Expression Omnibus. Accession number GSE154844.

## Author Contributions

MVR conceived the study, conducted all experiments, data analysis and the writing and editing of the manuscript. B-HL performed RNA-Seq analysis and contributed to the writing of the manuscript. EMSL and BHK contributed to the Western Blot experiments design, analysis, and the writing of the manuscript. CGMG contributed to the designing, analysis of the IVIS experiments and the writing of the manuscript. HI contributed to the experimental designing and the writing of the manuscript. MHL contributed to the designing and data analysis of all experiments and the writing and editing of the manuscript. All authors contributed to the article and approved the submitted version.

## Conflict of Interest

The authors declare that the research was conducted in the absence of any commercial or financial relationships that could be construed as a potential conflict of interest.
