# Supplementary material for: Chitin Attenuates Expression of Listeria monocytogenes Virulence Genes in vitro
Source: Front Microbiol. 2020 Dec 3;11:588906. doi: 10.3389/fmicb.2020.588906 (PMC7744463; doi:10.3389/fmicb.2020.588906)
Supplement: Supplementary file 1 [file Data_Sheet_1.PDF]

SUPPLEMENTARY INFORMATION

TABLES

SI Table 1. List of genes, primers and probes sequences employed to detect gene expression in this study.

Gene symbol	Primer/probe	5' → 3' SEQUENCE	Final concentration (nM)
LDH	Forward	ATGCTCGTAACGTCCATGGTT	300
	Reverse	GCTCCATGCTGGGAATTCTG	300
	TaqMan probe	6FAM-CATCCTTGGCGAACACGGCGA -TAMRA	100
RPOB	Forward	AGGCTTTGGTTCCGATCAAGAAATT	300
	Reverse	ACGCTTGAAAAAGACAACACTGACA	300
	TaqMan probe	6FAM-TGCACGTCACGTACTTCATAACCAGCA-BBQ	100
ACTA	Forward	GACCGACCAGCTATACAAGTGG	300
	Reverse	ACTATCCGATGATGCTATGGCTT	300
	TaqMan probe	6FAM-CTATCCGATGGCAATCCTGGATGACGA-BBQ	200

SI Table 2 – List of upregulated DEGs in *L. monocytogenes* EGD-e grown in CDM_{gly} in comparison to $CDM_{gly + chitin}$. DEGs are filtered by a log2 fold change (FC) cut-off ≤ -1 and a False Discovery Rate (FCR) < 0.05.

Log2 FC = logarithmic base 2-fold change **FDR** = False discovery ratio.

Gene id	Locus tag	Gene symbol	Gene function; description or name	log2 FC	FDR
CAD00365	lmo2287		Putative tape-measure protein	4.19	0.001
CAD00357	lmo2279		Holin [Bacteriophage A118]	4.15	0.003
CAD00362	lmo2284		Conserved hypothetical phage protein	4.12	< 0.001
CAD00361	lmo2283		Protein gp20 [Bacteriophage A118]	4.06	< 0.001
CAD00363	lmo2285		Protein gp18 [Bacteriophage A118]	4.05	< 0.001
CAD00364	lmo2286		Protein gp17 [Bacteriophage A118]	4.03	< 0.001
CAC99595	lmo1517		Nitrogen regulatory protein P-II	4.02	< 0.001
CAD00367	lmo2289		Conserved phage protein	4.01	< 0.001
CAD00359	lmo2281		Conserved hypothetical phage protein	3.99	0.007
CAC99594	lmo1516		Ammonium transporter	3.85	< 0.001
CAD00368	lmo2290		Ig-like virion protein	3.84	< 0.001
CAD00356	lmo2278	*lysA*	L-alanoyl-D-glutamate peptidase	3.83	< 0.001
CAD00369	lmo2291		Phage tail protein	3.82	< 0.001
CAD00366	lmo2288		Conserved phage protein	3.82	< 0.001
CAC98425	lmo0346	*tpiA2*	Probable triosephosphate isomerase 2	3.82	< 0.0001
CAD00376	lmo2298		Protein gp4 [Bacteriophage A118]	3.8	< 0.001
CAD00358	lmo2280	protein *gp23*	Conserved phage protein	3.77	< 0.001
CAD00360	lmo2282		Protein gp21 [Bacteriophage A118]	3.76	0.005
CAD00387	lmo2309		Unknown	3.75	0.003
CAD00373	lmo2295		Conserved phage protein	3.7	< 0.001
CAD00377	lmo2299		Putative portal protein [Bacteriophage A118]	3.68	< 0.001
CAD00374	lmo2296		Unknown	3.67	0.001
CAD00384	lmo2306		Unknown	3.66	< 0.001
CAD00375	lmo2297		Phage minor structural protein GP20	3.54	< 0.001
CAD00372	lmo2294		Protein gp9 [Bacteriophage A118]	3.48	< 0.001
CAD00370	lmo2292		Conserved phage protein	3.45	< 0.001
CAD00389	lmo2311		Unknown	3.41	0.005
CAD00371	lmo2293		Protein gp10 [Bacteriophage A118]	3.4	< 0.001
CAD00378	lmo2300		Putative terminase large subunit from Bacteriophage A118	3.36	< 0.001
CAD00386	lmo2308	*ssb2*	Single-stranded DNA-binding protein 2	3.3	0.003
CAD00924	lmo2711		Unknown	3.28	< 0.001
CAD00385	lin2401		Unknown	3.14	0.009
CAD00392	lmo2314		Unknown	3.06	0.012
CAD00382	lmo2304		Bacteriophage A118 gp65 protein	2.97	0.001
CAD00379	lmo2301		Unknown	2.96	< 0.001
CAC98426	lmo0347		Unknown	2.86	< 0.0001
CAD00383	lmo2305		Unknown	2.79	0.006
CAC98758	lmo0680	*fhlA*	Flagellar biosynthesis pathway protein	2.76	< 0.0001

CAD00381	lmo2303		Phage transcriptional regulator, ArpU family	2.66	< 0.001
CAD00390	lmo2312		Unknown	2.65	0.005
CAD00391	lmo2313		Unknown	2.45	0.011
CAD00393	lmo2315		Unknown	2.45	0.009
CAC98421	lmo0342		Transketolase	2.41	< 0.001
CAC98239	lmo0024		PTS system mannose/fructose/sorbose family IID component	2.26	0.001
CAD00515	lmo2437		Unknown	2.23	< 0.001
CAC99917	lmo1839	*pyrP*	Uracil permease	2.21	< 0.0001
CAD00394	lmo2316		Site-specific DNA-methyltransferase	2.19	0.002
CAC99737	lmo1659		Unknown	2.18	< 0.001
CAC98344	lmo0129		N-acetylmuramoyl-L-alanine amidase	2.18	< 0.001
CAD00861	lmo2648		Metal-dependent hydrolase, phosphotriesterase familyphosphotriesterase	2.17	< 0.0001
CAC98341	lmo0126		Unknown	2.16	< 0.001
CAC98342	lmo0127		Unknown	2.16	< 0.001
CAC98343	lmo0128		Putative phage holin protein	2.11	< 0.001
CAC98336	lmo0121		Phage tail protein	2.04	0.001
CAD00859	lmo2646		Unknown	2.02	< 0.0001
CAD00253	lmo2175	*fabgG*	3-ketoacyl-ACP reductase	2.01	< 0.001
CAD00864	lmo2651		PTS mannitol transporter subunit IIA	2.01	< 0.0001
CAD00349	lmo2271		Unknown	1.99	0.003
CAD00404	lmo2326		Unknown	1.98	0.013
CAC98340	lmo0125		Unknown	1.97	< 0.001
CAC98333	lmo0118	*lmaA*	Antigen A	1.95	0.001
CAC98759	lmo0681		Flagellar biosynthesis regulator FlhF	1.9	0.011
CAC98337	lmo0122		Phage tail family protein	1.9	0.001
CAC98549	lmo0470		Methyltransferase	1.86	< 0.001
CAD00354	lmo2276		Unknown	1.86	< 0.001
CAC98335	lmo0120		Phenylalanine racemase	1.85	0.001
CAC98339	lmo0124		CCA-adding enzyme	1.83	0.001
CAD00401	lmo2323		Gp43 [Bacteriophage A118]	1.82	0.003
CAC99916	lmo1838	*pyrB*	Aspartate carbamoyltransferase	1.82	< 0.001
CAC98338	lmo0123		Unknown	1.79	0.002
CAD00402	lmo2324		Phage antirepressor protein KilAC domain	1.77	0.005
CAD00395	lmo2317		DNA replication protein DnaD	1.76	0.005
CAD00396	lmo2318		ERF superfamily (Conserved domain)	1.75	0.021
CAC98332	lmo0117	*lmaB*	Antigen B	1.74	0.001
CAC98334	lmo0119		Segregation and condensation protein B	1.74	0.001
CAD00862	lmo2649	*ulaA*	PTS system ascorbate transporter subunit IIC	1.72	< 0.0001
CAC98240	lmo0025		Phosphoheptose isomerase	1.71	< 0.001
CAD00353	lmo2275		Protein gp28 [Bacteriophage A118]	1.69	< 0.001
CAC98427	lmo0348		Dihydroxyacetone kinase	1.69	< 0.001
CAD00664	lmo2586	*fdhF*	Formate dehydrogenase alpha chain	1.67	< 0.001
CAD00860	lmo2647		Creatinine amidohydrolase	1.64	< 0.0001
CAC98311	lmo0096	*manL*	PTS mannose transporter subunit IIAB	1.64	0.001

CAD00398	lmo2320		Unknown	1.62	0.005
CAD00863	lmo2650		PTS IIB ascorbate: subunit IIB of enzyme II (EII)	1.62	0.003
CAD00352	lmo2274		Protein gp29 [Bacteriophage A118]	1.61	0.001
CAD00403	lmo2325		Unknown	1.6	0.001
CAC98313	lmo0098		Putative PTS system, mannose-specific IID component	1.59	0.001
CAD00406	lmo2328		XRE family transcriptional regulator	1.59	0.004
CAD00405	lmo2327		Unknown	1.57	0.003
CAD00872	lmo2659		Ribulose-phosphate 3-epimerase	1.57	< 0.001
CAD00399	lmo2321		Protein gp45 of Bacteriophage A118	1.56	0.029
CAD00332	lmo2254		Xanthine/uracil/vitamin C permease	1.56	< 0.0001
CAC98423	lmo0344	*kduD*	2-deoxy-D-gluconate 3-dehydrogenase	1.55	< 0.001
CAC99596	lmo1518		Putative secreted protein	1.54	0.001
CAC99915	lmo1837	*pyrC*	Dihydroorotase	1.53	0.002
CAD00400	lmo2322		Gp44 [Bacteriophage A118]	1.5	0.029
CAD00347	lmo2269		IDEAL domain-containing protein	1.5	0.001
CAC98422	lmo0343	*tal*	Transaldolase	1.48	0.001
CAD00397	lmo2319		Unknown	1.47	0.006
CAD00873	lmo2660		Transketolase	1.46	< 0.001
CAC98312	lmo0097		PTS system mannose-specific IIC	1.44	0.003
CAD00351	lmo2273		Protein gp30 [Bacteriophage A118]	1.38	0.001
CAC99950	lmo1872		Methyltransferase	1.37	0.008
CAC98795	lmo0717		Transglycosylase	1.36	0.015
CAD00380	lmo2302		Unknown	1.3	0.003
CAC99914	lmo1836	*carA*	Carbamoyl-phosphate synthase small chain	1.29	0.012
CAC99059	lmo0981		Conserved domain of Fungal trichothecene efflux pump (TRI12)	1.26	< 0.001
CAC98270	lmo0055	*purA*	Adenylosuccinate synthetase	1.24	< 0.0001
CAC98975	lmo0897		Transporter -conserved domain Sulfate permease	1.24	0.002
CAD00350	lmo2272		Unknown	1.22	0.004
CAC99853	lmo1775	*purE*	N5-carboxyaminoimidazole ribonucleotide mutase	1.2	< 0.0001
CAD00577	lmo2499		Phosphate ABC transporter substrate-binding protein	1.19	0.012
CAD00886	lmo2673		Unknown	1.15	0.009
CAC98420	lmo0341		Universal stress protein family	1.15	0.003
CAC99075	lmo0997	*clpE*	Clp protease ATP-binding subunit	1.15	0.038
CAC98940	lmo0862		Oligo-1,6-glucosidase	1.13	< 0.001
CAC98754	lmo0676		Unknown	1.12	0.014
CAC98794	lmo0716	*fliI*	Flagellum-specific ATP synthase	1.12	0.023
CAD00160	lmo2082	*crcB2*	Putative fluoride ion transporter CrcB 2	1.12	0.006
CAC99918	lmo1840	*pyrR*	Bifunctional PyrR uracil phosphoribosyltransferase	1.12	< 0.001
CAD00450	lmo2372		ABC transporter ATP-binding protein	1.11	0.005
CAC99248	lmo1170		Unknown	1.11	0.001
CAD00575	lmo2497		Phosphate transport system permease protein PstA	1.09	0.008
CAD00102	lmo2024	*nadC*	Nicotinate-nucleotide pyrophosphorylase	1.09	0.003
CAC98796	lmo0718		Unknown	1.09	0.041
CAC99850	lmo1772	*purC*	Phosphoribosylaminoimidazole-succinocarboxamide synthase	1.09	< 0.0001

CAD00213	lmo2135		PTS system, Fru family, IIC component	1.08	0.011
CAD00532	lmo2454		Unknown	1.08	0.007
CAC98428	lmo0349		Unknown	1.07	0.003
CAC98424	lmo0345		Sugar-phosphate isomerase	1.07	0.004
CAC98778	lmo0700		Flagellar motor switch protein FliY	1.04	0.012
CAC98779	lmo0701		Eukaryotic translation initiation factor 3 subunit E	1.04	0.042
CAC98331	lmo0116	*lmaC*	Phage transcriptional regulator, ArpU family	1.02	0.006
CAC99849	lmo1771	*purS*	Phosphoribosylformylglycinamidine synthase subunit	1.01	0.003
CAC98652	lmo0573	*pbuG*	Guanine/hypoxanthine permease	1.01	0.003
CAC98792	lmo0714	*fliG*	Flagellar motor switch protein G	1.01	0.026
CAC98793	lmo0715	*fliH*	Flagellar assembly protein H	1.01	0.011

SI Table 3 – List of downregulated DEGs in *L. monocytogenes* EGD-e grown in CDM_{gly} in comparison to $CDM_{gly\ +\ chitin}$. DEGs are filtered by a log2 fold change (FC) cut-off ≤ -1 and a False Discovery Rate (FCR) < 0.05.

Gene id	Locus tag	Gene symbol	Gene function; description or name	log2 FC	FDR
CAD00191	lmo2113		Putative heme-dependent peroxidase	-1	< 0.001
CAC98410	lmo0195		ABC transporter	-1.01	0.006
CAD00492	lmo2414		Fe-S cluster assembly scaffold protein SufB	-1.01	0.026
CAD00588	lmo2510	*secA*	Protein translocase subunit SecA	-1.01	0.043
CAD00964	lmo2751		ABC transporter ATP-binding protein	-1.01	0.042
CAC98282	lmo0067		Dinitrogenase reductase ADP-ribosylation protein	-1.02	0.027
CAC98435	lmo0356		Oxidoreductase	-1.02	0.004
CAC98570	lmo0491	*aroD*	3-dehydroquinate dehydratase	-1.02	0.02
CAC98729	lmo0651		Transcriptional regulator	-1.02	0.02
CAC99715	lmo1637		Unknown	-1.02	0.006
CAD00016	lmo1938	*rpsA*	30S ribosomal protein S1	-1.02	0.016
CAD00266	lmo2188	*pepF*	Putative oligoendopeptidase F	-1.02	0.021
CAD00802	lmo0275		C-terminal part similar to B. subtilis ComEC	-1.03	0.017
CAC99101	lmo1023		Ktr system potassium uptake protein A	-1.03	0.04
CAD00983	lmo2770	*gshAB*	Glutathione biosynthesis bifunctional protein GshAB	-1.03	< 0.001
CAC98717	lmo0639		Transcripitonal regulator	-1.04	0.044
CAD00721	lmo2643		Unknown	-1.04	< 0.001
CAC99940	lmo1862		Unknown	-1.05	0.013
CAD01038	lmo2825	*serC*	Phosphoserine aminotransferase	-1.05	0.026
CAC98448	lmo0369		Probable transcriptional regulatory protein	-1.06	0.033
CAC99045	lmo0967		RelA/SpoT domain protein	-1.06	0.047
CAC99985	lmo1907	*dapB*	4-hydroxy-tetrahydrodipicolinate reductase	-1.06	0.026
CAD00870	lmo2657		Deoxyguanosinetriphosphate triphosphohydrolase-like protein	-1.06	0.003
CAD00808	lmo0281	*cfpL*	Transcriptional regulator	-1.07	0.037
CAC99856	lmo1778		ABC transporter ATP-binding protein	-1.07	0.047
CAC99982	lmo1904	*birA*	Bifunctional ligase/repressor BirA	-1.07	< 0.0001
CAC99984	lmo1906	*mgsA*	Methylglyoxal synthase	-1.07	0.032
CAD00527	lmo2449	*rnr*	Exoribonuclease RNase-R	-1.07	0.006
CAC99680	lmo1602		Hypothetical protein /General stress protein	-1.08	0.023
CAC99941	lmo1863		DegV domain-containing protein	-1.08	0.017
CAD00990	lmo2777		Bcr/CflA family drug resistance efflux transporter	-1.08	0.043
CAD01003	lmo2790	*parB*	Partition protein ParB homolg	-1.08	0.028
CAD01037	lmo2824		3-phosphoglycerate dehydrogenase	-1.08	0.001
CAC98496	lmo0417		2'-O-methyl transferase	-1.09	0.04
CAC98727	lmo0649		GntR family transcriptional regulator	-1.09	0.033
CAC99323	lmo1245		Putative cytosolic protein	-1.09	0.036
CAC99679	lmo1601		Putative general stress protein	-1.09	< 0.001
CAD00003	lmo1925	*hisC*	Histidinol-phosphate aminotransferase	-1.09	0.007
CAC99028	lmo0950		Alpha/beta hydrolase	-1.1	0.031

CAC99586	lmo1508		Sensor histidine kinase	-1.1	0.033
CAC99986	lmo1908		Nucleotide pyrophosphohydrolase	-1.1	0.044
CAD00526	lmo2448	*smpB*	SsrA-binding protein	-1.1	0.042
CAC98449	lmo0370		Unknown	-1.11	0.001
CAD00164	lmo2086		XRE family transcriptional regulator	-1.11	0.04
CAC98976	lmo0898		Protein YhgF	-1.12	0.035
CAD00723	lmo0196	*spoVG*	Putative septation protein SpoVG	-1.13	0.016
CAC98700	lmo0622		Putative membrane protein	-1.13	0.021
CAC99143	lmo1065		Unknown	-1.13	0.037
CAC99640	lmo1562	*nrdR*	Transcriptional repressor NrdR	-1.13	0.029
CAC99794	lmo1716		DNA-binding transcriptional regulator, AcrR family	-1.13	0.026
CAD00107	lmo2029		Unknown	-1.13	0.004
CAC98982	lmo0904		Unknown	-1.14	0.037
CAD00592	lmo2514		DegV domain-containing protein	-1.14	< 0.0001
CAD00593	lmo2515		Two-component response regulator DegU	-1.14	0.003
CAD00633	lmo2555		N-acetylglucosaminyl-phosphatidylinositol biosynthesis protein	-1.14	0.024
CAC98297	lmo0082		Unknown	-1.15	0.042
CAC99579	lmo1501		Unknown	-1.15	< 0.001
CAD00002	lmo1924	*tyrA*	Prephenate dehydrogenase	-1.15	0.012
CAD00324	lmo2246		Unknown	-1.15	0.028
CAD00580	lmo2502		Unknown	-1.15	0.026
CAD01015	lmo2802	*rsmG/gidB*	Ribosomal RNA small subunit methyltransferase G	-1.15	0.035
CAC99003	lmo0925		Putative membrane protein	-1.16	0.024
CAC99320	lmo1242		Unknown	-1.16	0.028
CAC99330	lmo1252		Unknown	-1.16	0.008
CAC99958	lmo1880		RNase HI	-1.16	0.02
CAD00569	lmo2491		Unknown	-1.16	0.012
CAD00943	lmo2730		Phosphatase	-1.16	0.009
CAC98235	lmo0020		GntR family transcriptional regulator	-1.17	0.007
CAC98698	lmo0620		Unknown	-1.17	0.018
CAC99418	lmo1340		Unknown	-1.17	0.019
CAD01017	lmo2804		Unknown	-1.17	0.002
CAC99142	lmo1064	*corA*	Putative magnesium/cobalt transporter, CorA family	-1.18	0.033
CAC99647	lmo1569	*FxsA*	Protein affecting phage T7 exclusion by the F plasmid	-1.18	0.022
CAC98283	lmo0068		Unknown	-1.19	0.026
CAC98828	lmo0750		Membrane protein	-1.19	0.02
CAC99673	lmo1595		GAF domain-containing protein	-1.19	0.036
CAD00073	lmo1995	*deoC/dra*	Deoxyribose-phosphate aldolase	-1.19	0.046
CAD00557	lmo2479		TPR domain-containing protein	-1.19	0.024
CAD00936	lmo2723		Unknown	-1.19	0.008
CAD00292	lmo2214		ABC transporter permease	-1.2	0.035
CAC99697	lmo1619	*daaA*	D-alanine aminotransferase	-1.21	0.045
CAD00435	lmo2357		Unknown	-1.21	0.003
CAD01002	lmo2789		Unknown	-1.21	0.001
CAC98659	lmo0580		Unknown	-1.22	0.011

CAC99135	lmo1057		L-lactate dehydrogenase	-1.22	0.001
CAD01033	lmo2820		Amino-terminal domain similar to transcription regulators	-1.22	0.021
CAC98534	lmo0455		PucR family transcriptional regulator	-1.23	0.033
CAC98692	lmo0614		N-acetyltransferase	-1.23	0.047
CAC99581	lmo1503		Unknown	-1.23	0.001
CAD00475	lmo2397		NifU protein: Fe-S cluster biogenesis protein	-1.23	0.004
CAD00497	lmo2419	*metN2*	Methionine import ATP-binding protein MetN 2	-1.23	0.046
CAC98590	lmo0511		GMP synthase	-1.24	0.04
CAC99029	lmo0951		Unknown	-1.24	0.028
CAD00074	lmo1996		DeoR family transcriptional regulator	-1.24	0.024
CAC98571	lmo0492		LysR family transcriptional regulator	-1.25	0.018
CAC99025	lmo0947		Hypothetical transport protein	-1.25	< 0.001
CAC99580	lmo1502	*yqgF*	Putative pre-16S rRNA nuclease	-1.25	< 0.0001
CAD00000	lmo1922		Unknown	-1.25	0.015
CAD00323	lmo2245		Unknown	-1.25	0.003
CAC98224	lmo0009		Spermidine acetyltransferase	-1.27	0.002
CAC99084	lmo1006		Aminotransferase	-1.27	0.037
CAC99657	lmo1579		Alanine dehydrogenase	-1.27	0.003
CAD00589	lmo2511	*hpf*	Ribosome hibernation promoting factor	-1.27	0.028
CAC98223	lmo0008		Cardiolipin synthase	-1.28	0.002
CAC98996	lmo0918	*licR*	Lichenan operon PTS system transcriptional antiterminator	-1.28	< 0.001
CAC98908	lmo0830	*fbp*	Fructose-1,6-bisphosphatase class 3	-1.29	< 0.0001
CAC98510	lmo0431		Acetyltransferase	-1.3	0.007
CAC98545	lmo0466		Unknown	-1.3	< 0.0001
CAC98564	lmo0485		Unknown	-1.3	0.01
CAC98589	lmo0510		Unknown	-1.3	0.037
CAD00865	lmo2652		PTS system transcriptional terminator	-1.3	< 0.001
CAC99104	lmo1026		LytR protein	-1.31	0.013
CAC98366	lmo0151		Unknown	-1.32	0.026
CAC98655	lmo0576		Hypothetical cell wall associated protein	-1.32	0.049
CAC98987	lmo0909		GntR family transcriptional regulator	-1.32	0.021
CAC99790	lmo1712		Multidrug resistance protein	-1.32	0.034
CAD00281	lmo2203		N-acetylmuramoyl-L-alanine amidase	-1.32	0.032
CAC99499	lmo1421		Glycine/betaine ABC transporter ATP-binding protein	-1.33	0.001
CAC98365	lmo0150		Unknown	-1.34	0.018
CAD00852	lmo0325		Transcriptional regulator	-1.34	0.007
CAC98988	lmo0910		Unknown	-1.34	0.003
CAC99346	lmo1268	*clpX*	ATP-dependent Clp protease ATP-binding subunit ClpX	-1.34	< 0.0001
CAC98498	lmo0419		Hypothetical protein	-1.35	0.02
CAC98918	lmo0840		MarR family transcriptional regulator	-1.35	0.034
CAC99136	lmo1058		Unknown	-1.35	< 0.001
CAC99347	lmo1269		Signal peptidase I	-1.35	0.044
CAC99792	lmo1714		Unknown	-1.35	0.018
CAD00596	lmo2518		LytR family transcriptional regulator	-1.35	0.022

CAC98617	lmo0538		N-acyl-L-amino acid amidohydrolase	-1.36	< 0.0001
CAC98693	lmo0615		Unknown	-1.36	0.042
CAC99500	lmo1422		Glycine/betaine ABC transporter permease	-1.36	0.001
CAD00198	lmo2120	*dacA*	Diadenylate cyclase	-1.36	0.011
CAC98321	lmo0106		Transcriptional regulator	-1.38	0.011
CAC98671	lmo0592		Unknown	-1.38	< 0.001
CAC98486	lmo0407		Unknown	-1.39	< 0.001
CAC99465	lmo1387		Pyrroline-5-carboxylate reductase	-1.39	0.021
CAD00197	lmo2119		Unknown	-1.39	< 0.001
CAD00496	lmo2418		ABC transporter permease	-1.39	0.035
CAC98494	lmo0415		Endo-1,4-beta-xylanase	-1.4	0.01
CAC98840	lmo0762	*hflX*	GTPase HflX	-1.42	0.006
CAD00521	lmo2443		Unknown	-1.42	0.035
CAD00644	lmo2566	*lipL*	Lipoyl-[GcvH]:protein N-lipoyltransferase	-1.42	< 0.001
CAC98431	lmo0352		DeoR family transcriptional regulator	-1.43	< 0.0001
CAC98986	lmo0908		Predicted PurR-regulated permease PerM	-1.45	0.044
CAC99022	lmo0944		HesB-like protein	-1.45	0.009
CAD00963	lmo2750	*pabB*	Para-aminobenzoate synthase component 1	-1.45	0.001
EBG00000016619	lmo0410		Unknown	-1.46	0.043
CAD00918	lmo2705		Soluble KIT ligand	-1.46	< 0.0001
CAC98522	lmo0443		LytR family transcriptional regulator	-1.48	< 0.001
CAC99329	lmo1251		Fnr/Crp family transcriptional regulator	-1.48	0.018
CAC98616	lmo0537		N-carbamyl-L-amino acid amidohydrolase	-1.51	0.011
CAD00044	lmo1966		5-bromo-4-chloroindolyl phosphate hydrolysis protein	-1.51	0.026
CAD00547	lmo2469		APA family basic amino acid/polyamine antiporter	-1.52	0.029
CAC98626	lmo0547		DeoR family transcriptional regulator	-1.53	0.001
CAD00230	lmo2152		Thioredoxin	-1.53	< 0.0001
CAC99040	lmo0962	*lemA*	Listeria epitope LemA	-1.54	0.005
CAD00545	lmo2467		Chitin-binding protein	-1.54	< 0.001
CAC98490	lmo0411		Phosphoenolpyruvate synthase	-1.55	0.002
CAC98605	lmo0526		Transcriptional regulator	-1.55	0.001
CAC98707	lmo0629		Unknown	-1.55	0.014
CAC99085	lmo1007		Hypothetical 8.22 kDa protein	-1.55	0.045
CAD00488	lmo2410		Unknown	-1.55	0.003
CAD00297	lmo2219	*prsA2*	Foldase protein PrsA	-1.57	< 0.001
CAC98376	lmo0161		Unknown	1.6	< 0.001
CAC98921	lmo0843		Unknown	1.6	0.023
CAC99054	lmo0976		Unknown	1.6	0.001
CAC99026	lmo0948		Transcriptional regulator	-1.61	0.006
CAC99999	lmo1921		UPF0302 protein	-1.61	< 0.0001
CAC99055	lmo0977		Unknown	-1.62	0.009
CAD00254	lmo2176		TetR family transcriptional regulator	-1.62	0.017
CAD00600	lmo2522		LysM peptidoglycan-binding domain-containing protein	-1.63	0.033
CAC99562	lmo1484	*comEA*	Competence protein ComEA	-1.64	0.04
CAD00682	lmo2604		Unknown	-1.64	< 0.001

CAD00789	lmo0262	*inlG*	Internalin G	-1.65	0.011
CAD00231	lmo2153		Similar to flavodoxin	-1.65	< 0.0001
CAC98667	lmo0588		DNA photolyase	-1.68	< 0.0001
CAD00971	lmo2758	*guaB*	Inosine-5'-monophosphate dehydrogenase	-1.68	< 0.0001
CAC98515	lmo0436		DNA binding transcription factor activity	-1.7	0.023
CAD00233	lmo2155		Ribonucleoside-diphosphate reductase	-1.7	< 0.0001
CAC98315	lmo0100		Unknown	-1.73	0.009
CAC98383	lmo0168		AbrB family transcriptional regulator	-1.73	0.019
CAD00232	lmo2154	*nrdF*	Ribonucleoside-diphosphate reductase subunit beta	-1.79	< 0.0001
CAC99791	lmo1713		Rod shape-determining protein MreB	-.18	0.037
CAD00564	lmo2486		Unknown	-2.05	0.006
CAD00517	lmo2439		Lipoprotein	-2.14	0.024
CAD00192	lmo2114		Similar to ABC transporter (ATP-binding protein)	-2.2	< 0.0001
CAC99033	lmo0955		Unknown	-2.29	0.025
CAD00193	lmo2115		ABC transporter permease	-2.29	0.002
CAC99032	lmo0954		Unknown	-2.3	0.021
CAD00646	lmo2156		Unknown	-2.62	0.024
CAC98513	lmo0434	*inlB*	Internalin B	-4.55	< 0.0001
CAC98512	lmo0433	*inlA*	Internalin A	-4.88	< 0.0001
CAD00727	lmo0200	*prfA*	Listeriolysin positive regulatory protein	-5.62	< 0.0001
CAC99864	lmo1786	*inlC*	Internalin C	-5.99	< 0.0001
CAD00734	lmo0207	*orfZ*	Putative lipoprotein	-6.14	< 0.0001
CAD00729	lmo0202	*hly*	Listeriolysin O	-6,9	< 0.0001
CAD00733	lmo0206	*orfX*	Unknown	-7.15	< 0.0001
CAD00730	lmo0203	*mpl*	Zinc metalloproteinase	-7.52	< 0.0001
CAD00728	lmo0201	*plcA*	1-phosphatidylinositol phosphodiesterase	-7.58	< 0.0001
CAC98916	lmo0838	*uhpT*	Hexose-6-phosphate:phosphate antiporter	-7.85	< 0.0001
CAD00732	lmo0205	*plcB*	Phospholipase C	-8.49	< 0.0001
CAD00731	lmo0204	*actA*	Actin assembly-inducing protein	-8.7	< 0.0001

SI Table 4 – List of differentially expressed ncRNAs in *L. monocytogenes* EGD-e grown in CDM_{gly} in comparison to $CDM_{gly + chitin}$. ncRNAs are filtered by a log2 fold change (FC) cut-off ≤ -1 and a False Discovery Rate (FCR) < 0.05.

ncRNA	symbol	log2 FC	FDR
rli47		5,27	0.001
rli12	ssrS-6S	3,73	< 0.001
rli19	ssrA	3,66	< 0.001
rli20	SRP	3,24	< 0.001
rli17	LhrA	2,69	0.002
rli18	rliG	2,62	0.002
rli15	rnpB	2,44	0.002
rli140		1,27	0.01
rli98		1,2	0.004
rli37		-1,16	0.03
rli72		-1,19	0.01
rli41		-1,23	0.007
rli24		-1,51	0.005
rli113		-1,69	0.01
rli96		-1,92	0.008
rli77		-5,09	< 0.0001
rli74		-9,02	< 0.0001

FIGURES

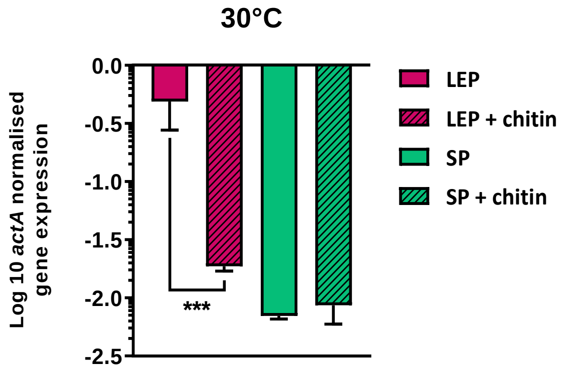

SI Fig. 1- Comparison of *actA* expression normalised by geometric mean (*rpoB/ldh*) using qRT-PCR of *L. monocytogenes* EGD-e grown in CDM_{gly} and $CDM_{gly + chitin}$ at 30°C in LEP and SP. The expression of *actA* is upregulated in LEP and significantly repressed by chitin. Comparisons were made between growth phase groups with chitin against without.

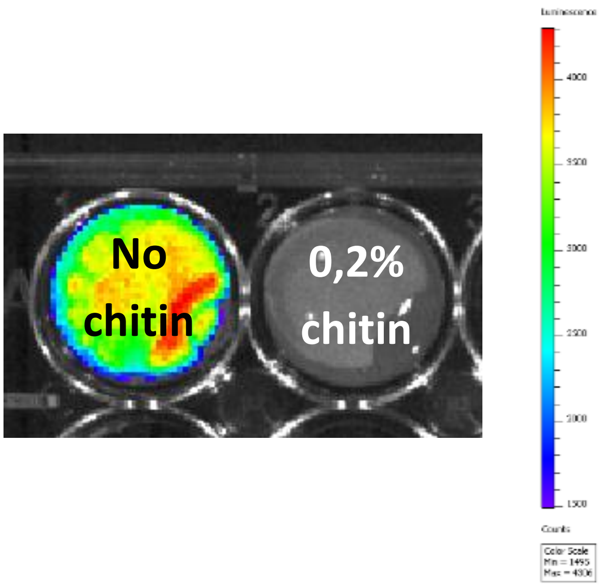

SI Fig. 2 – No bioluminescence signal was retrieved when chitin was present overnight in EGD-e::pPL2*lux*-P_{hly} mutant cultures grown in DMEM. The image was taken for an integrating time of 2 min.

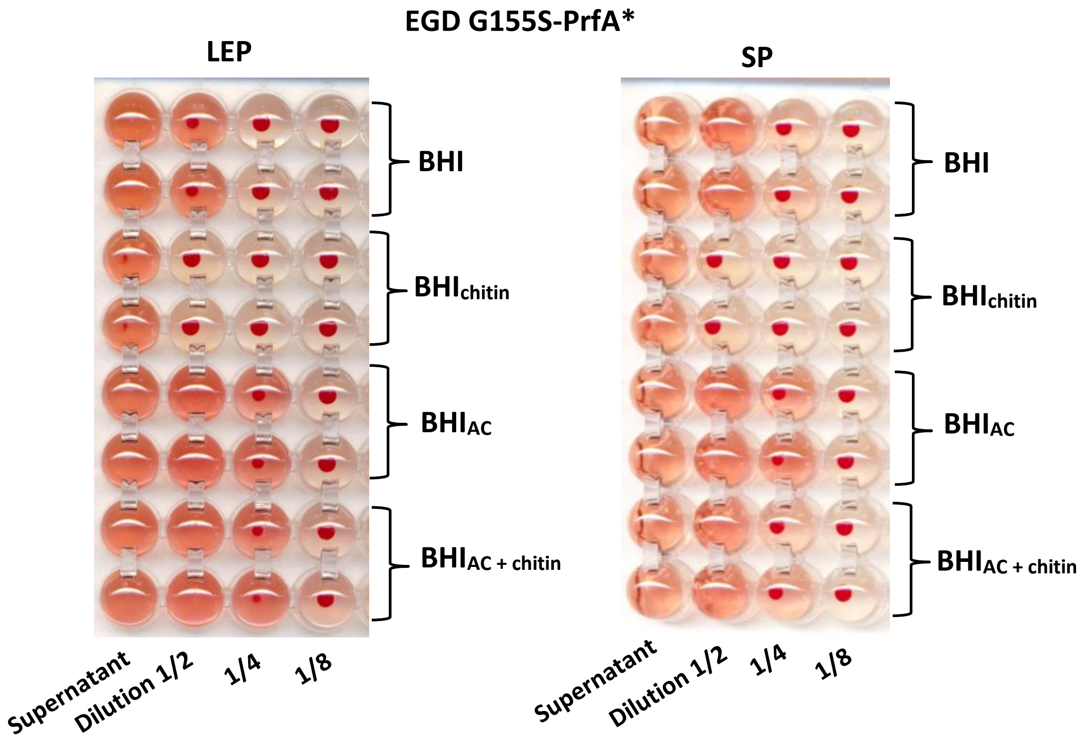

SI Fig. 3 – Haemolytic activity of an EGD G155S-PrfA* mutant is not significantly inhibited when grown in rich media in either LEP or SP.
